# SIV Genome-Wide Pyrosequencing Provides a Comprehensive and Unbiased View of Variation within and outside CD8 T Lymphocyte Epitopes

**DOI:** 10.1371/journal.pone.0047818

**Published:** 2012-10-24

**Authors:** Austin L. Hughes, Ericka A. Becker, Michael Lauck, Julie A. Karl, Andrew T. Braasch, David H. O’Connor, Shelby L. O’Connor

**Affiliations:** 1 Department of Biological Sciences, University of South Carolina, Columbia, South Carolina, United States of America; 2 Wisconsin National Primate Research Center, University of Wisconsin, Madison, Wisconsin, United States of America; 3 Department of Pathology and Laboratory Medicine, University of Wisconsin, Madison, Wisconsin, United States of America; University of Pittsburgh Center for Vaccine Research, United States of America

## Abstract

Deep sequencing technology is revolutionizing our understanding of HIV/SIV evolution. It is known that acute SIV sequence variation within CD8 T lymphocyte (CD8-TL) epitopes is similar among MHC-identical animals, but we do not know whether this persists into the chronic phase. We now determine whether chronic viral variation in MHC-identical animals infected with clonal SIV is similar throughout the entire coding sequence when using a sensitive deep sequencing approach. We pyrosequenced the entire coding sequence of the SIV genome isolated from a unique cohort of four SIVmac239-infected, MHC-identical Mauritian cynomolgus macaques (MCM) 48 weeks after infection; one MCM in the cohort became an elite controller. Among the three non-controllers, we found that genome-wide sequences were similar between animals and we detected increased sequence complexity within 64% of CD8-TL epitopes when compared to Sanger sequencing methods. When we compared sequences between the MHC-matched controller and the three non-controllers, we found the viral population in the controller was less diverse and accumulated different variants than the viral populations in the non-controllers. Importantly, we found that initial PCR amplification of viral cDNA did not significantly affect the sequences detected, suggesting that data obtained by pyrosequencing PCR-amplified viral cDNA accurately represents the diversity of sequences replicating within an animal. This demonstrates that chronic sequence diversity across the entire SIV coding sequence is similar among MHC-identical animals with comparable viral loads when infected with the same clonal virus stock. Additionally, our approach to genome-wide SIV sequencing accurately reflects the diversity of sequences present in the replicating viral population. In sum, our study suggests that genome-wide pyrosequencing of immunodeficiency viruses captures a thorough and unbiased picture of sequence diversity, and may be a useful approach to employ when evaluating which sequences to include as part of a vaccine immunogen.

## Introduction

One major challenge facing HIV vaccine researchers is the unrelenting ability of HIV to mutate in the presence of vaccine-elicited host immune responses. This relationship leads to the selection of immune escape variants that ultimately thwart attempts by host immune responses to control viral replication. Approximately 60% of sequence variants detected in immunodeficiency viruses are selected by host CD8 T lymphocyte (CD8-TL) responses and these polymorphisms are a major factor contributing to worldwide HIV sequence diversity [1]–[7].

There has been much debate over which epitope sequences should be included in a CD8-TL based HIV vaccine. Eliciting CD8-TL responses targeting immunodominant epitopes that select for immune escape variants in conserved regions may rapidly reduce virus viability during an infection [8]. Alternately, CD8-TL responses targeting conserved epitopes that do not escape may preserve functional epitope-specific T cell responses and sustain long-term control [9]. Examining sequence variation within a few isolated epitopes as a way to choose vaccine antigens is inherently biased. Instead, a comprehensive picture of variation within known CD8-TL epitopes across the entire viral genome is necessary to inform vaccine design.

Recent advances in sequencing technologies have revolutionized our ability to characterize immunodeficiency virus populations. For the past 10 years, Sanger sequencing of bulk PCR amplicons or a limited number of cDNA clones was used to detect high frequency viral variants. Recently, the development of single genome amplification has helped inform studies of transmitted/founder viruses [10]. Unfortunately, these tools may miss sequence variants that likely have an important biological role and thus underestimate viral population diversity. In contrast, deep sequencing immunodeficiency viruses can be used to approximate the frequency of each variant, characterize the linkage of multiple polymorphisms within a single virus, and identify low frequency variants that previously went undetected [11]–[15]. By using this technology, we can obtain a more detailed view of the viral population to determine the extent of variability that can be tolerated within each epitope among individuals with a known genetic background. This improves our understanding of host-virus relationships and can inform the design of a CD8-TL based HIV vaccine.

Non-human primates infected with simian immunodeficiency virus (SIV) are a valuable model in which to study virus-specific CD8-TL responses and the corresponding selection of viral sequence variants [16]. Although commonly studied Indian rhesus macaques can be infected with clonal SIV, the diverse immunodominance hierarchies found among these typically MHC-disparate animals can confound studies aimed at characterizing the timing and sequence of variants that accumulate in CD8-TL epitopes [6], [17]. In contrast to Indian rhesus macaques, groups of MHC-identical Mauritian cynomolgus macaques (MCM) can be readily selected for challenge with clonal SIV [18], [19]. Using this unique system, we can determine (a) whether viral sequence evolution is predictable when controlling for both host and viral genetics, and (b) the extent of sequence variation across all described CD8-TL epitopes in animals with a specific MHC genotype.

In this study, we take advantage of a group of four SIVmac239 infected MHC-identical MCM, one of which became an elite controller (EC). We find that the sequences detectable in the three MHC-matched non-controllers were similar to each other, but different from the EC. Additionally, we find increased complexity in 64% of the CD8-TL epitopes present in the virus populations in the non-controllers, further emphasizing that sequence diversity is not limited to a few CD8-TL epitopes. Lastly, we find that sequences obtained by directly pyrosequencing viral RNA are similar to those observed by pyrosequencing PCR-amplified viral cDNA, suggesting that pyrosequencing approaches that employ initial PCR generate sequence data that accurately reflects the replicating virus population. Overall, our data provides compelling evidence that genome-wide deep sequencing of HIV/SIV viruses is a valuable and accurate approach to comprehensively characterize the diversity of sequences within a viral population.

## Methods

### Animal Care and Use and Ethics Statement

Animals were cared for the by the Wisconsin National Primate Research Center (WNPRC) according to protocols approved by the University of Wisconsin Research Animal Resources Center review committee (Protocol #G00363). The four animals were infected with SIVmac239 as part of other studies in David O’Connor’s laboratory. Plasma was collected from these animals during the course of infection and we obtained permission to use the samples in this study. The sequencing analyses relevant to this study were performed using frozen plasma samples collected 48 weeks after infection.

The enclosures for each animal had at least 4.3, 6.0, or 8.0 square feet of floor space and measured 30, 32, or 36 inches high, according to AWA regulations. Each enclosure also contained a tubular PVC or stainless steel perch, and was equipped with a horizontal or vertical sliding door, an automatic water lixit, and a stainless steel feed hopper.

The WNPRC uses a nutritional plan for its nonhuman primate colony that is based on recommendations published by the National Research Council. The animals were fed twice daily a fixed formula, extruded dry diet (2050 Teklad Global 20% Protein Primate Diet) with enough carbohydrate, energy, fat, fiber (10%), mineral, protein, and vitamin content. The feeding strategy for each animal was tailored to its age and physical condition. Supplemental fruits, vegetables, and other edible objects (e.g. nuts, cereals, seed mixtures, yogurt, peanut butter, popcorn, marshmallows, etc.) were added to the dry diet to provide variety and to inspire species-specific foraging behaviors.

Additional foraging opportunities, food enrichment, human-to-monkey interaction, structural enrichment, and manipulanda were provided to the animals by the Behavioral Management Unit of the WNPRC to promote species-typical behavior and psychological well-being. The objects selected for enrichment were chosen to minimize the chance of pathogen transmission between animals and between animals and care staff.

All animals were alive when the plasma samples were collected for this project, but only one animal was still alive at the time this manuscript was submitted. Animals were evaluated for signs of pain, distress, and illness by observing appetite, stool quality, activity level, physical condition, etc. by staff at the WNPRC at least twice per day. If any of those parameters appeared abnormal, a member of the WNPRC veterinary staff was notified and appropriate clinical care was provided to the animal. If a WNPRC veterinarian believed that an animal developed an untreatable or incurable condition that caused significant pain or distress, then the WNPRC veterinarian recommended an animal for euthanasia. Several SIV disease progression factors were also considered (e.g. inappetance, weight loss, opportunistic infection, etc.). CY0163, CY0164, and CY0166 were euthanized between the time the samples were collected for this study and the time this manuscript was submitted. Euthanasia was performed by an intravenous (IV) overdose of sodium pentobarbital or equivalent as approved by a clinical veterinarian, preceded by ketamine. The euthanasia procedures complied with the American Veterinary Medical Association’s Guidelines of Euthanasia.

### Genome-wide SIV Pyrosequencing by RT-PCR

Pyrosequencing the entire SIV genome was performed, essentially, as previously described [12]. Briefly, viral RNA was isolated with the QIAamp MinElute virus spin kit (Qiagen, Valencia, CA). Viral RNA was reverse transcribed and four amplicons spanning the entire SIV genome were generated with the Superscript™ III One-Step RT-PCR system with Platinum® Taq High Fidelity (Invitrogen, Carlsbad, CA) and SIV-specific primers. PCR products were purified with the Qiagen MinElute Gel Extraction Kit (Qiagen) and quantified using the Quant-IT dsDNA HS Assay Kit (Invitrogen). For viruses isolated from CY0163, CY0164, CY0166, and an SIVmac239 stock, 12.5 ng of each purified amplicon were combined together, but for viruses isolated from CY0165, the amount of template ranged from 5 to 20 ng per amplicon due to its low viral load. Libraries were prepared using the Nextera™ DNA Sample Prep Kit (Roche Titanium-Compatible) (Epicentre, Madison, WI) and 10 bp multiplex identifier (MID) tags were added. Tagged products were cleaned twice using Agencourt AMPure XP beads (Beckman Coulter Genomics, Danvers, MA) and the products were quantified using the Quant-IT dsDNA HS Assay Kit (Invitrogen) and the Agilent High Sensitivity DNA kit (Agilent Technologies, Santa Clara, CA). Pyrosequencing was performed with a Roche/454 GS Junior instrument and Titanium shotgun chemistry, according to the manufacturer’s protocols (454 Life Sciences, Brandford, CT).

### Genome-wide SIV Sequencing by Direct Pyrosequencing

Direct pyrosequencing of viral RNA was performed, essentially, as previously described [20]. Briefly, plasma (1 ml) was centrifuged at 5,000×g at 4°C for 5 min with subsequent filtration of the supernatant through a 0.45-µm filter (Millipore, Billerica, MA, USA). Viral RNA was isolated as above, except that carrier RNA was omitted. Eluted RNA was treated with DNase I (DNA-free, Ambion, Austin, TX, USA), and double stranded DNA was generated using the Superscript® double-stranded cDNA Synthesis kit (Invitrogen, Carlsbad, CA, USA) primed with random hexamers. The Agencourt Ampure XP system (Beckman Coulter, Brea, CA, USA) was used to purify DNA. An MID-tagged library was generated with approximately 1 ng of DNA, and then pyrosequenced as described above.

### Characterizing Percent Variation from Inoculum at Each Nucleotide Position

Sequence reads were base called with Roche base caller version 2.5 p1 and converted to FASTQ files. Nucleotide sequence alignments were then performed using a suite of online tools available at a local installation of *Galaxy* [21], [22]. We first trimmed Roche/454 adaptor sequences, MID tags, and transposon sequences from FASTQ files. Low quality bases (quality <18) were masked with an “N” and reads were then mapped to SIVmac239 (Accession # M33262) using LASTZ at a 90% identity threshold [23]. We used SAM tools to calculate the percent variation at each nucleotide position, excluding nucleotide sites that were masked [24]. Data was imported into a local installation of LabKey software [25] for storage.

### Characterizing Amino Acid Variation within CD8-TL Epitopes

Adapter, MID, and transposon sequences were initially trimmed from FASTQ sequences in *Galaxy*. Low quality sequences (quality <18) were masked with an “N” before all sequences were aligned to SIVmac239 (Accession #M33262) using LASTZ at 90% identity to create a SAM output. Custom scripts were used to extract sequences spanning each CD8-TL epitope in FASTA format. These sequences were then aligned to SIVmac239 in CodonCode Aligner (CodonCode Corporation, Deadham, MA) at 80% identity using “Local alignments.” Sequences were trimmed to within three nucleotides upstream and downstream of each epitope. Sequences were then reassembled into contigs with 100% identity using “End to end alignments.” In cases where the coverage was high, the number of maximum successive failures was set to 500 or 1000 to ensure that sequences assembled. Sequences were manually removed from contigs if they contained an “N” in a position that was not degenerate. The amino acid sequences of each contig and the number of reads within a contig were compiled. Variant sequences that were present at less than 1% frequency or were detectable at any frequency in the inoculum were categorized as “Other.”

### Calculation of Sequence Diversity

For a given SIV sample, we estimated synonymous nucleotide diversity (*π_S_*) within the sample by computing the pairwise average number of synonymous differences and dividing the latter quantity by the number of synonymous sites from the inoculum sequence. Likewise, we estimated nonsynonymous nucleotide diversity (*π_N_*) by computing the pairwise average number of nonsynonymous differences and dividing by the number of nonsynonymous sites from the inoculum sequence. Following Nei and Gojobori [26], estimates of *π_S_* and *π_N_* were corrected for multiple hits by the Jukes-Cantor method. In preliminary analyses, we found that these procedures yielded results very similar to the results obtained by the Nei and Gojobori (1986) method, using pairwise deletion in the MEGA4 program [27]. Only variants present at 1% or greater were included in these analyses, as other studies suggest this is a reasonable threshold for inclusion [28].

We computed the Pearson correlation coefficient between the proportion variant at SNP sites that were variable in different host MCM. In these analyses we included only sites that showed a different nucleotide from the inoculum in at least one host. We computed the correlations separately for synonymous and nonsynonymous SNP sites. We included in these calculations only SNPs that were synonymous in all reading frames (N = 553) and SNPs that were nonsynonymous in all reading frames (n = 501). Again, only variants present at 1% or greater were included in these analyses.

### Construction of Phylogenetic Trees

We constructed phylogenetic trees from individual sequencing reads covering codons 367 to 404 of the Gag protein. Trees were constructed by the neighbor-joining method [29] on the basis of the uncorrected proportion of nucleotide difference, with pairwise deletion of sites with undetermined nucleotides or gaps postulated by the alignment.

## Results

### Genome-wide Pyrosequencing of SIV Replicating in MHC-matched M3/M3 MCM

We pyrosequenced the entire SIV coding sequence replicating at 48 weeks post-infection in four MHC-matched SIVmac239-infected MCM and an SIVmac239 stock, using methods previously described [12]. These four animals were homozygous for the M3 MHC haplotype (CY0163, CY0164, CY0165, and CY0166) [18], [19]. Three animals (CY0163, CY0164, and CY0166) had relatively high viral loads at one year post-infection (Table S1). Although MHC-identical, CY0165 became an elite controller, with viral loads at one year after infection approaching the limit of detection. We obtained an average of 54,565 reads for each genome (Table S1). For all animals except CY0165, the number of theoretical templates was greater than the average coverage, minimizing concerns about PCR template resampling.

### Increased Intrahost Viral Diversity in Non-controller Animals

With this high-resolution view of the viral population, we wanted to determine whether there was a relationship between intrahost viral sequence diversity and the effective population size of the virus. We compared both the synonymous (*π_S_*) and nonsynonymous (*π_N_*) nucleotide diversity within the entire viral population from all four animals at non-overlapping sites with greater than 1% variation. We found that both *π_S_* and *π_N_* were significantly lower in the virus population isolated from CY0165 than from the other three MHC-identical animals (Table 1), suggesting that the viral population in CY0165 is less diverse than the viral populations in the animals with uncontrolled viral replication. Additionally, we found that *π_S_* was greater than *π_N_* in the three non-controllers, but not in CY0165, an observation that is consistent with the hypothesis that purifying selection is more effective in larger population sizes [30].

**Table 1:** Synonymous (*π_S_*) and nonsynonymous (*π_N_*) nucleotide diversity in non-overlapping coding regions of SIV populations.

Host	*π_S_* ± S.E.	*π_N_* ± S.E.
CY0165	0.0011±0.0008	0.0011±0.0005
CY0163	0.0082±0.0022**	0.0036±0.0009*
CY0164	0.0101±0.0025***	0.0039±0.0009**
CY0166	0.0072±0.0021*	0.0038±0.0009**
Mean (all except CY0165)	0.0085±0.0009***	0.0038±0.0001***

* *π_S_* or *π_N_* significantly different from corresponding value for CY0165 (P<0.05; Z-test).

** *π_S_* or *π_N_* significantly different from corresponding value for CY0165 (P<0.01; Z-test).

*** *π_S_* or *π_N_* significantly different from corresponding value for CY0165 (P<0.001; Z-test).

We were concerned that the low intrahost viral sequence diversity observed in CY0165 might be an artifact of template resampling, as the theoretical number of templates was often far less than the coverage at an individual epitope. To address this concern, we examined the sequence diversity among individual reads from the same region of the genome that included a high frequency SNP. We identified a common mutation in the Mafa-A1*063-restricted Gag_386–394_GW9 epitope: P390S. This mutation could be attributed to a C to T mutation present at >99% frequency at nucleotide position 2476. We identified 375 reads spanning amino acids 367 to 404 in Gag. We detected a total of 58 polymorphic sites within this region. A phylogenetic tree (Figure S1) based on the proportion nucleotide difference among the sequences, with pairwise deletion of sites with alignment gaps or undetermined nucleotides, showed a complex branching pattern. Even though the number of reads exceeded the number of theoretical templates, this analysis suggested that numerous independent viral templates were sampled. We performed a similar analysis with the 90 sequencing reads spanning Gag 367 to 404 from CY0163, and we found an equally complex similar branching pattern (Figure S2) even though this group of sequences had a higher level of nucleotide sequence diversity. Based on our calculation of theoretical templates, it is likely that there was some template resampling of viruses from CY0165, but the similar branching pattern observed among sequences isolated from both animals mitigate this concern. Template resampling is an inherent concern when sequencing viruses from elite controllers and may have impacted our results, but we think there is no *a priori* reason to expect more or less variation in a small virus sample. It is possible that random sampling of viral RNA in the initial RT-PCR reaction could lead to the detection of *different* variants in multiple sequencing replicates from the same elite controller at the same time point. In this study, this possibility was also unlikely because the variants we detected within CD8-TL epitopes by deep sequencing matched those obtained by Sanger sequencing high frequency variants in bulk PCR amplicons.

### Improved Detection of Sequence Variation within CD8-TL Epitopes

**Figure 1:**
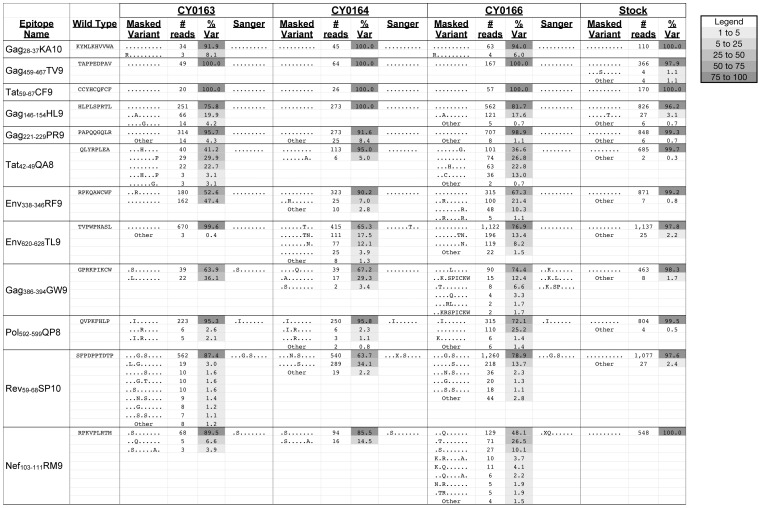
Variation in CD8-TL epitopes in virus populations from non-controller M3/M3 MCM and an SIVmac239 stock. Amino acid variation detectable by pyrosequencing in 12 CD8-TL epitopes in viruses isolated from three M3/M3 MCM (CY0163, CY0164, and CY0166) is compared to variation previously reported by Sanger sequencing bulk PCR amplicons [18]. Amino acid variants detectable within an SIVmac239 stock were characterized here and elsewhere [34]. Sequences that match the inoculum are represented with a “.”. Mixed populations are represented with an “X”. The number of high quality reads with each specific sequence is shown. The total number of reads for each epitope can be found in Table S2. The frequency of reads with a specific amino acid sequence is shown as “% Var.” The sequences labeled as “other” include variants that were individually present at less than 1% and variants that were also detected in the inoculum, which included the A2V mutation in Gag_221–229_PR9. The relative shading of each box reflects the frequency of reads, as indicated in the legend.

It has been demonstrated that pyrosequencing SIV/HIV provides a more comprehensive picture of sequence diversity in epitopes known to rapidly escape dominant CD8 T cell responses [11], [13], [31], but we wanted to test the hypothesis that pyrosequencing would detect variants within CD8-TL epitopes that were thought to be largely resistant to diversification. Twelve CD8-TL epitopes restricted by MHC class I alleles on the M3 MHC haplotype have been recently described, but only four of these epitopes (Gag_386–394_GW9, Pol_592–599_QP8, Rev_59–68_SP10, and Nef_103–111_RM9) have been shown to consistently accumulate sequence variants [32]–[34]. We quantified the accumulation of amino acid variants in all 12 epitopes from all three non-controllers at 48 weeks post infection that were detectable by pyrosequencing, as described in the Methods. We excluded all sequences that did not have high quality coverage across the *entire* epitope. Coverage between epitopes was highly variable (Table S2). Some epitopes contain low quality homopolymeric tracts that are known to be difficult to sequence with Roche/454 chemistry [28]. We calculated the percent of reads with a given amino acid sequence and compared the sequences to variants detected in an SIVmac239 stock (Figure 1 and [34]). All high quality variant sequences present at a frequency of less than 1% or matching sequences present in the inoculum were categorized as “Other.” Only two variants in the inoculum were found at a frequency of 1–5%, and none were present at a frequency greater than 5% (Figure 1), giving us confidence that most of the detected variants were not attributable to PCR errors. Therefore, the relative frequency of a variant was the most objective way to establish a cutoff for inclusion, even if there were only a small number of high-quality reads covering a specific CD8-TL epitope.

We compared amino acid variation detectable by pyrosequencing with previously reported amino acid variation detectable by Sanger sequencing bulk PCR amplicons prepared from viruses isolated from the same animals at the exact same time point [18]. Sanger sequencing of bulk PCR amplicons classified 24 epitopes as wild type, whereas 12 epitopes had accumulated sequence variants ([18] and Figure 1). When we applied the analyses described above to our pyrosequencing data, we detected amino acid variants in 12 of the 24 epitopes (50%) that were previously classified as wild type. The frequency of these variants was as high as 67%, demonstrating that Sanger sequencing is insufficient for detection of sequence variants, including some that are relatively common. We also found a more complex population of amino acid variants in 11 of the 12 epitopes that were previously classified as either a single static variant or a mixed variant. In one exceptional example, we readily detected an amino acid insertion in the Mafa-A1*063-restricted Gag_386–394_GW9 epitope in the virus population isolated from CY0166; this insertion was more difficult to characterize by bulk Sanger sequencing because individual sequence reads could not be isolated. Overall, the increased complexity of variation within known CD8-TL epitopes is remarkable and demonstrates the enormity of viral sequence diversity that has been overlooked by previous sequencing technologies.

To determine whether the increased variation we detected within epitopes was consistent with the hypothesis that it was driven by immune selection, rather than improved detection of nucleotide variation, we calculated the *π_N_* and *π_S_* within each epitope and in the remainder of the genes (Table 2 and Table S3). We found that *π_N_* was greater than *π_S_* across the 12 epitopes with variation detectable by pyrosequencing, but not by Sanger sequencing. In contrast, the value of *π_N_* was less than *π_S_* across the remainders of the six genes (*gag*, *pol*, *env*, *tat*, *ref*, and *nef*). This data further suggests that the increased variation that we specifically detected within epitopes by pyrosequencing was driven by host immune responses.

**Table 2:** Synonymous (π_S_) and nonsynonymous (π_N_) nucleotide diversity in variant epitopes from CY0163, CY0164, and CY0166.

Region	π_S_ ± S.E.	π_N_ ± S.E.
Epitopes with variation detected only by pyrosequencing	0.0043±0.0020	0.0188±0.0048^a^
Epitopes with variation detected by Sanger & pyrosequencing	0.0022±0.0009	0.0288±0.0059^b^
Remainders	0.0080±0.0009	0.0045±0.0007^a^

Paired t-tests of the hypothesis that π_S_ = π_N_: ^a^P<0.05; ^b^P<0.01.

See Table S3 for the value of nucleotide diversity for individual epitopes and remainders.

### Initial PCR Amplification of Viral cDNA Accurately Reflects Sequences in the Total Virus Population

**Figure 2:**
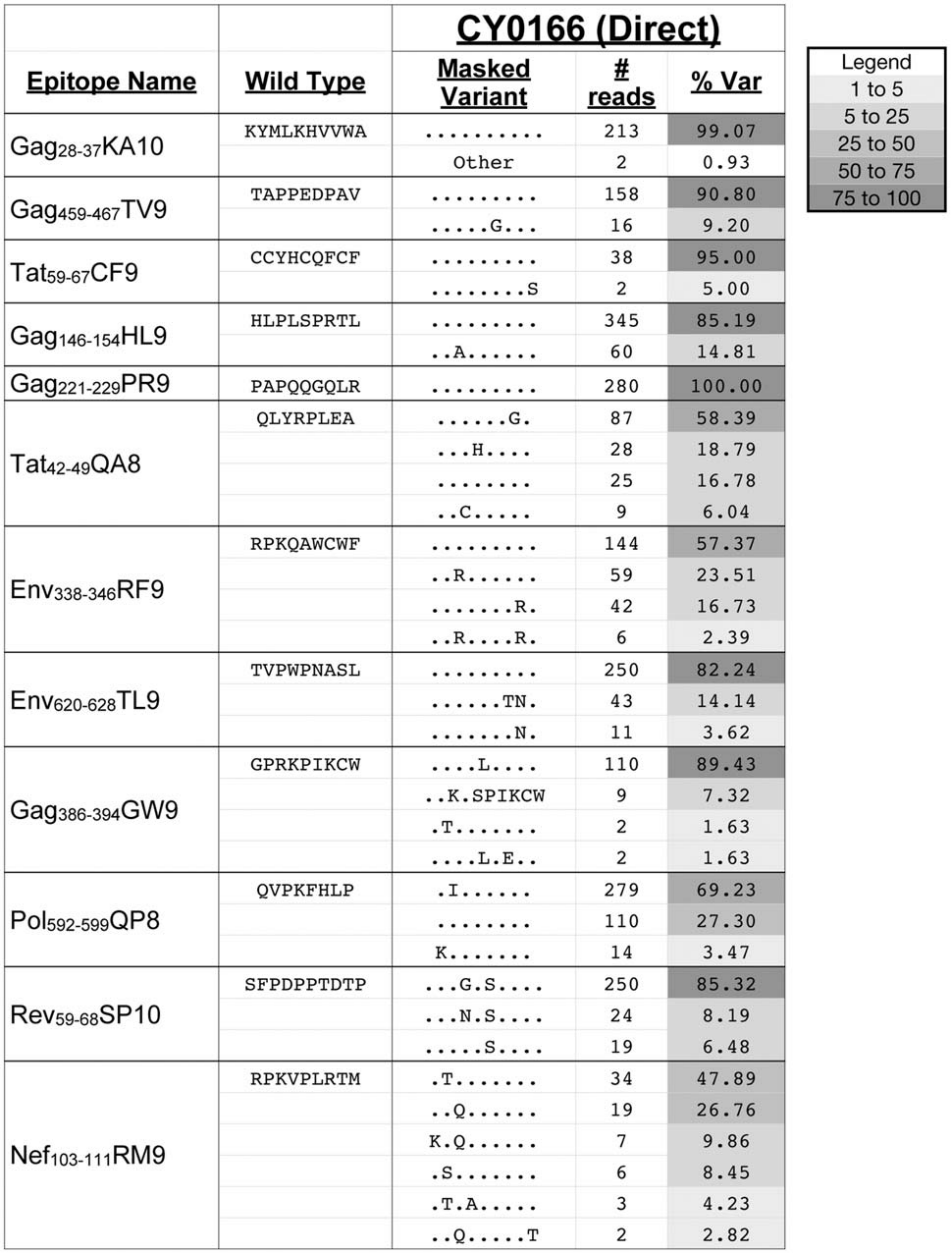
Sequence variants in CD8-TL epitopes are detectable by directly pyrosequencing virus populations isolated from CY0166. Amino acid variation detected by directly pyrosequencing the virus population in CY0166 is shown for all 12 CD8-TL epitopes. Sequences that match the inoculum are represented with a “.”. The number of high quality reads with each specific sequence is shown. The total number of reads for each epitope can be found in Table S2. The frequency of reads with a specific amino acid sequence is shown as “% Var.” The sequences labeled as “other” include variants that were individually present at less than 1% and variants that were also detected in the inoculum. The relative shading of each box reflects the frequency of reads, as indicated in the legend.

Our method to pyrosequence SIV employs an initial RT-PCR step to create enough templates needed for sequencing. Unfortunately, this added step has the potential to introduce sequencing artifacts. To confirm that the RT-PCR accurately amplified sequences in the replicating population of virus, we compared this approach to one where we directly pyrosequenced viral RNA. Direct pyrosequencing of RNA viruses was previously used to isolate and sequence novel RNA viruses from plasma in the absence of initial PCR [20]. In this study, we directly pyrosequenced viral RNA from CY0166 at 48 weeks post-infection and compared it to the data obtained using the RT-PCR method. We compared 503 sites that differed from the inoculum (with a percent variant of at least 1%) in one or both approaches. These sites were unambiguously synonymous (N = 259) or nonsynonymous (N = 244) in all reading frames. The correlation between percent variant in the two samples was 0.975 (P<0.001) for all sites, 0.938 (P<0.001) for synonymous sites only, and 0.981 (P<0.001) for nonsynonymous sites only. We also examined the CD8-TL epitope sequences as described above, and we found the diversity of sequences was similar in all epitopes, except for Nef_103–111_RM9 (Figure 2). The differences at this epitope may be attributable to the low coverage, such that a slight bias in the primers used to perform the initial amplification may have dramatically altered the hierarchy of detectable variants. Although there are some advantages to direct pyrosequencing, this approach can only be used to sequence viruses from animals with high viral loads and it is more expensive, making it less feasible for widespread use. Importantly, our results demonstrate that the two approaches yield largely similar data, suggesting that data obtained by pyrosequencing PCR-amplified viral cDNA provides a reasonably accurate reflection of the sequences replicating in the total virus population.

### Reduced Sequence Diversity within CD8-TL Epitopes Replicating in an MHC-identical Elite Controller

**Figure 3:**
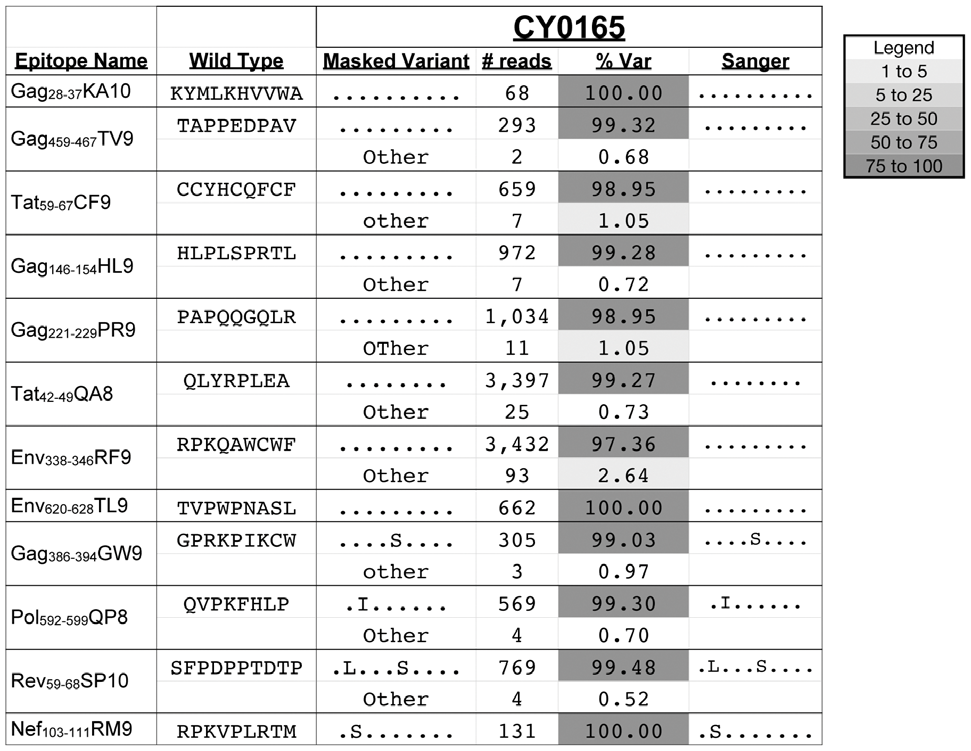
Variation in CD8-TL epitopes in a virus population from an M3/M3 elite controller. Amino acid variation detected by pyrosequencing in 12 CD8-TL epitopes in viruses isolated from CY0165 is compared to variation previously reported by Sanger sequencing bulk PCR amplicons [18]. Sequences that match the inoculum are represented with a “.”. The number of high quality reads with each specific sequence is shown. The total number of reads for each epitope can be found in Table S2. The frequency of reads with a specific amino acid sequence is shown as “% Var.” The sequences labeled as “other” include variants that were individually present at less than 1% and variants that were also detected in the inoculum. The relative shading of each box reflects the frequency of reads, as indicated in the legend.

We then examined sequence diversity in the same 12 CD8-TL epitopes in the virus population isolated from the EC, CY0165. At all 12 epitopes, we found that amino acid variation detected by Sanger sequencing bulk PCR amplicons matched the amino acid variation detected by pyrosequencing (Figure 3). Although there were some low frequency variants (“Other”), many of these fell below our 1% threshold, or they were also detected in the SIVmac239 stock. We found that nucleotide diversity (*π_N_* and *π_S_*) in all 12 epitope regions in viruses isolated from CY0165 was significantly lower than in viruses isolated from the non-controllers (Table 3). Given this reduced viral diversity, it is not surprising that both Sanger sequencing and pyrosequencing yielded the same information. One caveat to this data set is that coverage at several epitopes in the CY0165 virus population was higher than the theoretical number of templates (Table S1). Even though this suggests that some resampling likely occurred and the absolute value of *π_N_* and *π_S_* may be an underestimation, the ratio of *π_N_* to *π_S_* should be unaffected. Thus, the smaller *π_N_* to *π_S_* ratio in CY0165 is consistent with the hypothesis that less effective purifying selection occurred in this smaller virus population, and correspondingly, fewer sequence variants were observed.

**Table 3:** Synonymous (π_S_) and nonsynonymous (π_N_) nucleotide diversity in epitope and non-epitope regions from all animals.

Host	Epitope		Non-Epitope	
	π_S_ ± S.E.	π_N_ ± S.E.	π_S_ ± S.E.	π_N_ ± S.E.
CY0165	0.0011±0.0006	0.0014±0.0005^c^	0.0006±0.0003^d^	0.0006±0.0002^c^
Others	0.0035±0.0012	0.0206±0.0037^b^	0.0069±0.0009	0.0044±0.0008
All	0.0029±0.0009	0.0158±0.0033^a^	0.0053±0.0009	0.0035±0.0007

Paired t-tests of the hypothesis that π_S_ = π_N_; ^a^ P<0.05; ^b^ P<0.01.

Nucleotide diversity is analyzed in all 12 epitope and non-epitope regions of the SIV genomes isolated from all four animals (mean values by gene). T-tests of the hypothesis that π_S_ or π_N_ for CY0165 equals the corresponding value for the other animals: ^c^ P<0.01; ^d^ P<0.001.

In viruses isolated from all four animals, *π_N_* within the epitope regions was greater than *π_N_* in the non-epitope regions (Table 3). This data indicates that escape mutations accumulated in all four animals, but the number of epitopes acquiring mutations was drastically different in the controller and non-controller animals (Figures 1 and 3). In the elite controller, mutations accumulated in only four epitopes, three of which are known to be targeted by acute CD8-TL responses. This data suggests that studies exploring sequence variation in larger cohorts of SIV-infected MHC-matched MCM might shed light on whether replication is controlled more effectively in virus populations that fail to acquire mutations in epitopes targeted by subdominant CD8-TL responses.

### Unique Sequence Variation Detected in an Elite Controller

We had an opportunity to determine whether sequence variation in viruses replicating in an EC matched the SNPs present in viruses replicating in his MHC-matched non-controlling counterparts. The correlation coefficient between the proportion variant at genome-wide non-overlapping SNP sites was used to measure the similarity of viral sequence evolution among the four hosts (Table 4). At nonsynonymous sites, the proportion variant was strongly positively correlated among the three non-controllers (P<0.001 in each case) (Table 4). The proportion variant at nonsynonymous sites in CY0165 was significantly positively correlated with that in CY0163, but was not significantly correlated with that in CY0164 or in CY0166 (Table 4). However, the correlations between the non-controllers were all significantly greater (P<0.001; Bonferroni-corrected) than the highest correlation (r = 0.152) between CY0165 and any non-controller. At synonymous sites, the proportion variant was significantly positively correlated among the three non-controllers (Table 4), but these correlations were lower and significantly different (P<0.001 in each case) from the corresponding correlations among nonsynonymous SNP sites (Table 4). The proportion variant at synonymous SNP sites was not significantly correlated between any of the non-controllers and CY0165 (Table 4). We only examined non-overlapping SNPs, such that SNPs that were synonymous in one of the 9 main SIV reading frames but nonsynonymous in another one of the 9 main SIV reading frames were excluded from analyses (Table 4). Therefore, the observed correlation among non-controllers at synonymous sites likely reflects higher mutability of certain synonymous sites, unappreciated cryptic epitopes [35], or synonymous variants hitchhiking with selectively favored nonsynonymous variants. Taken together, these results indicate that a different set of polymorphic sites was found in SIV replicating in the EC CY0165 than those seen in the other hosts, and that the tendency toward shared variants among CY0163, CY0164, and CY0166 was particularly marked in the case of nonsynonymous sites. This implies that the virus populations replicating in non-controllers had similar sequences, while the viruses replicating in CY0165 were somewhat different, even though all four animals have the same MHC genotype and they were infected with the same viral sequence.

**Table 4:** Correlation coefficients between proportion variant at SNP sites in SIV from different hosts.

		CY0163	CY0164	CY0166
Synonymous^1^	CY0165	−0.041	−0.037	−0.034
	CY0163		0.140*	0.307***
	CY0164			0.130*
Nonsynonymous^2^				
	CY0165	0.152*	0.120	0.079
	CY0163		0.617*** †	0.653*** †
	CY0164			0.567*** †

1 Only sites synonymous in all reading frames and showing one or more differences from the inoculum in at least one host (N = 451 sites) were included.

2 Only sites nonsynonymous in all reading frames and showing one or more differences from the inoculum in at least one host (N = 420 sites) were included.

* Significance of correlation coefficient: P<0.05 (Bonferroni-correction for multiple testing).

** Significance of correlation coefficient: P<0.01 (Bonferroni-correction for multiple testing).

*** Significance of correlation coefficient: P<0.001 (Bonferroni-correction for multiple testing).

† The correlation for nonsynonymous sites was significantly different (P<0.001 in each case; Bonferroni-correction for multiple testing) from the corresponding correlation for synonymous sites.

Additional results were likewise indicative of a tendency toward unique nonsynonymous variants in SIV replicating in CY0165 but toward shared variants in viruses replicating in the other three monkeys. The majority (65.5%) of nonsynonymous SNPs present at a frequency of 1% or greater were unique to SIV replicating in CY0165, whereas in all other hosts, the majority (52.3%–59.7%) of nonsynonymous SNPs were shared with at least one other host (P = 0.001, Table 5). By contrast, no such difference was seen at synonymous SNPs between viruses circulating in CY0165 and that in other hosts (Table 5).

**Table 5:** Median proportion variant at sites shared or unique to the SIV from each host.

	Host	Shared		Unique	
		N (%)	Prop. variant	N (%)	Prop. variant
Synonymous	CY0165	9 (36.0%)^1^	0.1069	16 (64.0%)	0.0478
	CY0163	88 (43.1%)	0.0264	116 (56.9%)	0.0155***
	CY0164	100 (44.8%)	0.0193	123 (55.2%)	0.0145**
	CY0166	78 (49.4%)	0.0196	80 (50.6%)	0.0164
Non-synon.	CY0165	30 (34.5%)^2^	0.0288	57 (65.5%)	0.0261
	CY0163	112 (58.9%)	0.0473	78 (41.1%)	0.0159***
	CY0164	102 (52.3%)	0.0578	93 (47.7%)	0.0174***
	CY0166	89 (59.7%)	0.0728	60 (40.3%)	0.0187***

1 The proportions of shared and unique synonymous SNPs did not differ significantly among hosts; χ^2^ = 2.32; 3 d.f.; N.S.

2 The proportions of shared and unique nonsynonymous SNPs differed significantly among hosts; χ^2^ = 15.71; 3 d.f.; P = 0.001.

** Mann-Whitney test of the hypothesis that the median proportion variant of shared SNPs equaled that of unique SNPs: P<0.01.

*** Mann-Whitney test of the hypothesis that the median proportion variant of shared SNPs equaled that of unique SNPs: P<0.001.

Even though this is a relatively small sample size, data from both Tables 4 and 5 indicate that chronic sequence diversity is similar among MHC-identical animals that are infected with a clonal virus and who have a similar outcome. Detection of a unique set of sequences replicating in CY0165 further supports the hypothesis that purifying selection was less efficient in the animal with a smaller effective population size. Therefore, host MHC-genetics and infecting viral sequence are not sufficient to predict the chronic sequences of the viral population in animals with different viral load trajectories.

## Discussion

Recent advances in sequencing technologies are revolutionizing our understanding of complex viral populations. Although immunodeficiency viruses have long been considered exceptionally diverse, it was previously too difficult to reasonably capture this diversity across the entire coding sequence. With deep sequencing tools, such as Roche/454pyrosequencing, the enormity of this diversity is elucidated, allowing researchers to ask new questions about viral sequence evolution and viral escape from host immune responses.

As HIV vaccines that elicit CD8-TL responses are developed, it is important to understand the potential variability of defined epitope sequences. Are there low frequency variants in epitopes that were previously thought unable to accumulate escape mutations? Are there highly mutable epitopes that can sustain a large diversity of variants? Are there ultra-conserved epitopes that cannot tolerate any variability? Can viral populations in ECs sustain an extensive diversity of viral sequences? Understanding the answers to these questions is necessary to formulate HIV vaccine antigens designed to elicit CD8-TL responses targeting epitope sequences with specified characteristics.

In this study, we wanted to determine whether Sanger sequencing underestimated the diversification within CD8-TL epitope sequences in the chronic phase across the entire SIV coding sequence, including those epitopes that were previously thought to be relatively fixed. We chose to examine viral populations isolated from a unique cohort of MHC-identical MCM 48 weeks after SIVmac239 infection. We expected similar immunodominance hierarchies among this group of MHC-identical animals, and thus we expected the pattern of genome-wide sequence variants would be similar. We then compared viral sequence variation in 12 known CD8-TL epitopes detected by pyrosequencing and previously reported by Sanger sequencing bulk PCR amplicons. We found extensive sequence variability by pyrosequencing that previously went undetected by Sanger sequencing. Immune responses were examined by IFNγ-ELISPOT to many of these epitopes in these animals, but responses were not always detectable by this assay ([32] on-clonal technologieso and unpublished observations). Furthermore, detection of a response by IFNγ-ELISPOT did not necessarily predict whether variants accumulated within an epitope. This observation further underscores the need to examine sequence variation across multiple CD8-TL epitopes in a manner that is not biased by immunology assays that measure host responses at a single time point.

The different sensitivity of the two sequencing methods is best highlighted in three epitopes: Tat_42–49_QA8 in CY0163 and CY0166 and Gag_386–394_GW9 in CY0164. These three epitopes were previously classified as wild type by Sanger methods, but less than 30% of sequences were classified as wild type by pyrosequencing (Figure 1). Nonetheless, these discordant results at such highly mutable epitopes highlight that even relatively high frequency variants are not always confidently called by Sanger methods. This is consistent with another report suggesting that Sanger sequencing of bulk PCR amplicons detects only 3/4 of the variants present at a frequency of 20% or greater [36]. Therefore, these notable examples highlight that data obtained by Sanger consensus sequencing can still overlook key information about immune escape that is gained by pyrosequencing SIV.

Variants detectable by pyrosequencing, but not by Sanger sequencing, were found at frequencies ranging from 1.1% to 67.2%. Although the direct impact of these low frequency variants is unclear, similarly low frequency antiretroviral drug resistant variants associated with a poor clinical outcome have been detected in HIV+ individuals [37]. These observations suggest that low frequency variants could be biologically relevant, and similar studies of low frequency immune escape variants and their relationship to virological failure is warranted.

In this study, we found that the sequences of viruses replicating in MHC-identical non-controllers during the chronic phase were remarkably similar. This data suggests that when both host MHC genetics and infecting viral sequence are controlled, viral diversity follows a similar pattern in animals with similar outcomes. This approach could now be used in future studies to better understand immunodeficiency virus evolution in animals that share no MHC alleles, one MHC allele, or a full MHC haplotype. Additionally, this approach could also be used to determine whether viral evolution is similar in animals that are MHC-identical, but infected with a non-clonal swarm SIV inoculum.

Even though we studied a relatively small cohort of animals, our unique group included an MHC-identical EC. Great efforts have been made to determine whether the sequences of viruses found in HIV+ and SIV+ ECs are distinct from the sequences of viruses found in HIV+ and SIV+ non-controllers. Although mutations exist within CD8-TL epitopes in virus populations isolated from HIV+ ECs, these viruses tend to harbor fewer mutations both within and outside CD8-TL epitopes than observed in virus populations isolated from non-controllers [38]–[40]. Additionally, fewer distinct mutations have been detected within CD8-TL epitopes of SIV+ ECs than in SIV+ progressors who share a single MHC class I allele [41], [42]. Our data from SIV+ MHC-identical macaques is consistent with observations from both of these previous studies. We found fewer SNPs within the virus population from CY0165. We also observed reduced nucleotide diversity both inside and outside CD8-TL epitopes. By making these similar observations with an exceptionally small cohort of MCM, it suggests that future studies that capitalize upon the exquisite sensitivity of pyrosequencing or other deep sequencing methods may identify several nuances of viral sequence signatures associated with containment of immunodeficiency virus replication.

### Conclusions

In this group of animals with tightly controlled host and viral genetics, we use genome-wide pyrosequencing technologies to examine the consistency of viral sequence diversity during chronic infection. We also examined variation that accumulated within 12 CD8-TL epitopes at the same time from each animal, and we provide evidence that immune escape from both acute and chronic CD8-TL responses is more extensive than previously thought. We also found that genome-wide viral variation was similar in MHC-identical non-controllers, but distinct in the one MHC-matched elite controller in our cohort. We also found that data obtained by pyrosequencing PCR-amplified viral cDNA was similar to that obtained by directly pyrosequencing viral RNA. Therefore, we propose that pyrosequencing the full coding sequence of SIV provides a comprehensive and unbiased picture of the spectrum of variants within the viral population, thus shedding light on which host and viral factors contribute to sequence diversity and the mutability of each CD8-TL epitope.

## Supporting Information

Figure S1 **Phylogenetic tree of viral sequences from CY0165 spanning codons 367 to 404 of the Gag protein.**
(TIF)

Figure S2 **Phylogenetic tree of viral sequences from CY0163 spanning codons 367 to 404 of the Gag protein.**
(TIF)

Table S1 **Metrics for pyrosequencing viruses isolated from the four animals in this study.**
(DOCX)

Table S2 **Coverage at each epitope.**
(DOCX)

Table S3 **Synonymous (π_S_) and nonsynonymous (π_N_) nucleotide diversity for each epitope region and the remainders of each gene for CY0163, CY0164, and CY0166.**
(DOCX)
