# Supplementary figures and images for: SIV Genome-Wide Pyrosequencing Provides a Comprehensive and Unbiased View of Variation within and outside CD8 T Lymphocyte Epitopes
Source: PLoS One. 2012 Oct 24;7(10):e47818. doi: 10.1371/journal.pone.0047818 (PMC3480401; doi:10.1371/journal.pone.0047818)

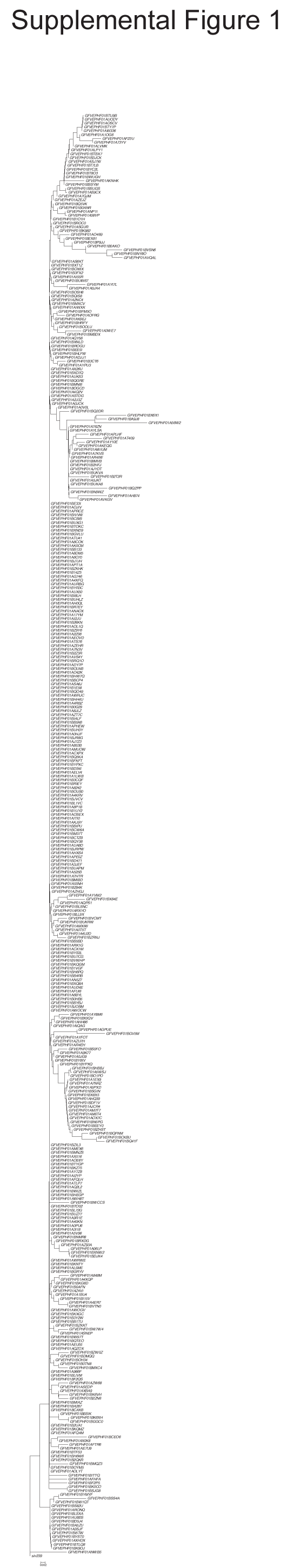

Supplement: Figure S1 — Phylogenetic tree of viral sequences from CY0165 spanning codons 367 to 404 of the Gag protein. (TIF) [file pone.0047818.s001.tif]

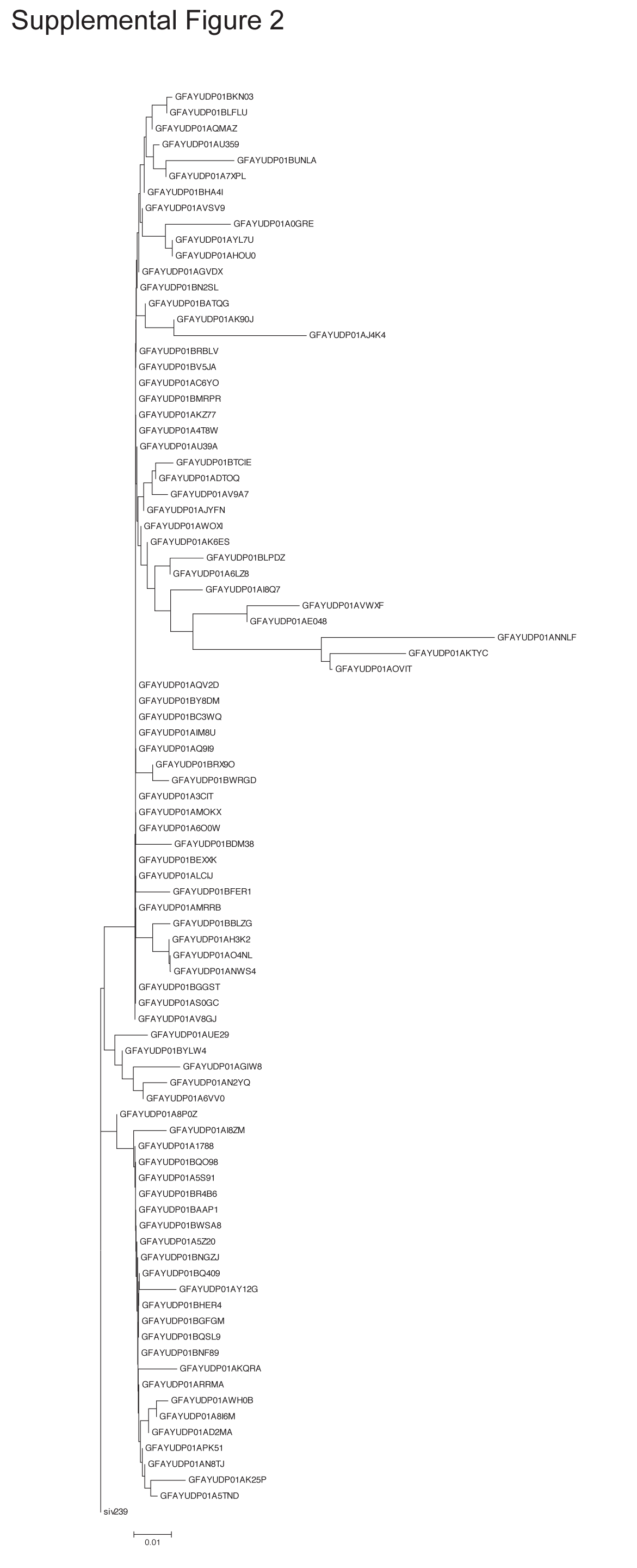

Supplement: Figure S2 — Phylogenetic tree of viral sequences from CY0163 spanning codons 367 to 404 of the Gag protein. (TIF) [file pone.0047818.s002.tif]
