# Supplementary material for: SIV Genome-Wide Pyrosequencing Provides a Comprehensive and Unbiased View of Variation within and outside CD8 T Lymphocyte Epitopes
Source: PLoS One. 2012 Oct 24;7(10):e47818. doi: 10.1371/journal.pone.0047818 (PMC3480401; doi:10.1371/journal.pone.0047818)
Supplement: Table S1 — Metrics for pyrosequencing viruses isolated from the four animals in this study. (DOCX) [file pone.0047818.s003.docx]

Table S1: Metrics for pyrosequencing viruses isolated from the four animals in this study

| **Animal ID** | **Viral load** | **# of reads** | **Average coverage** | **Theoretical templates** |
| --- | --- | --- | --- | --- |
| CY0163 | 4.94E+06 | 46,090 | 478 | 370,500 |
| CY0164 | 3.38E+06 | 56,091 | 559 | 253,500 |
| CY0166 | 3.92E+06 | 66,463 | 979 | 294,000 |
| CY0165 | 2.58E+03 | 35,913 (A,B,D) & 39,971 (C) | 1607 | 258 (A,B,D) & 469 (C) |

The viral load (copies/ml plasma) at 48 weeks post-infection and the number of sequencing reads are shown. Virus populations from CY0165 were sequenced from two different viral RNA preparations in two separate runs: amplicons A, B, and D were prepared and sequenced in a run separate from amplicon C. The average coverage is the average number of high quality A, C, T, or G bases at each site throughout the viral genome. To determine the number of theoretical templates, we assumed maximal recovery of viral RNA from the QIAamp MinElute column and divided this by the volume of elution buffer to determine the concentration of our viral RNA. We multiplied the viral RNA concentration by the volume of viral RNA used in each RT-PCR reaction to calculate the number of theoretical templates.
