# Supplementary material for: SIV Genome-Wide Pyrosequencing Provides a Comprehensive and Unbiased View of Variation within and outside CD8 T Lymphocyte Epitopes
Source: PLoS One. 2012 Oct 24;7(10):e47818. doi: 10.1371/journal.pone.0047818 (PMC3480401; doi:10.1371/journal.pone.0047818)
Supplement: Table S2 — Coverage at each epitope. (DOCX) [file pone.0047818.s004.docx]

Table S2: Coverage at each epitope

| **Epitope** | **CY0163** | **CY0164** | **CY0166** | **CY0165** | **Stock** | **CY0166 Direct** |
| --- | --- | --- | --- | --- | --- | --- |
| Gag_28-37_KA10 | 37 | 45 | 67 | 68 | 110 | 215 |
| Gag_459-467_TV9 | 49 | 64 | 167 | 295 | 374 | 174 |
| Tat_59-67_CF9 | 20 | 26 | 57 | 666 | 170 | 40 |
| Gag_146-154_HL9 | 331 | 273 | 688 | 979 | 859 | 405 |
| Gag_221-229_PR9 | 328 | 298 | 715 | 1045 | 854 | 280 |
| Tat_42-49_QA8 | 97 | 119 | 276 | 3422 | 687 | 149 |
| Env_338-346_RF9 | 342 | 358 | 468 | 3525 | 878 | 251 |
| Env_620-628_TL9 | 673 | 636 | 1459 | 662 | 1162 | 304 |
| Gag_386-394_GW9 | 61 | 58 | 121 | 308 | 471 | 123 |
| Pol_592-599_QP8 | 234 | 261 | 437 | 573 | 808 | 403 |
| Rev_59-68_SP10 | 643 | 848 | 1596 | 773 | 1104 | 293 |
| Nef_103-111_RM9 | 76 | 110 | 268 | 131 | 548 | 71 |

The table shows the number of total reads with complete high quality sequence data for each CD8-TL epitope in each population of viruses. In general, coverage at each epitope was consistent between genomes sequenced by RT-PCR of viral RNA, except for Tat_59-67_CF9, Tat_42-49_QA8, and Env_338-346_RF9 in viruses replicating in CY0165. Sequences replicating in CY0165 covering these three epitopes were included in a single amplicon run which had 39,971 reads, whereas 35,913 sequences covering the remainder of the genome were collected separately. We also sequenced viruses present in CY0166 by directly pyrosequencing RNA (CY0166 Direct), and the coverage at each of these epitopes was different from sequences collected using the RT-PCR method.
