# Supplementary material for: SIV Genome-Wide Pyrosequencing Provides a Comprehensive and Unbiased View of Variation within and outside CD8 T Lymphocyte Epitopes
Source: PLoS One. 2012 Oct 24;7(10):e47818. doi: 10.1371/journal.pone.0047818 (PMC3480401; doi:10.1371/journal.pone.0047818)
Supplement: Table S3 — Synonymous (πS) and nonsynonymous (πN) nucleotide diversity for each epitope region and the remainders of each gene for CY0163, CY0164, and CY0166. (DOCX) [file pone.0047818.s005.docx]

Table S3. Synonymous (π_S_) and nonsynonymous (π_N_) nucleotide diversity for each epitope region and the remainders of each gene for CY0163, CY0164, and CY0166

| **Variation only detected by pyrosequencing*** | | | |
| --- | --- | --- | --- |
| Epitope | Animal | π_S_ | π_N_ |
| Gag_28-37_KA10 | CY0163 | 0.0000 | 0.0064 |
| Gag_146-154_HL9 | CY0163 | 0.0145 | 0.0181 |
| Tat_42-49_QA8 | CY0163 | 0.0000 | 0.0612 |
| Env_338-346_RF9 | CY0163 | 0.0000 | 0.0233 |
| Tat_42-49_QA8 | CY0164 | 0.0000 | 0.0047 |
| Env_338-346_RF9 | CY0164 | 0.0294 | 0.0080 |
| Gag_386-394_GW9 | CY0164 | 0.0000 | 0.0481 |
| Gag_28-37_KA10 | CY0166 | 0.0000 | 0.0055 |
| Gag_146-154_HL9 | CY0166 | 0.0030 | 0.0138 |
| Tat_42-49_QA8 | CY0166 | 0.0105 | 0.0589 |
| Env_338-346_RF9 | CY0166 | 0.0000 | 0.0246 |
| Env_620-628_TL9 | CY0166 | 0.0037 | 0.0305 |

| **Variation detected by Sanger and pyrosequencing** | | | |
| --- | --- | --- | --- |
| Epitope | Animal | π_S_ | π_N_ |
| Gag_386-394_GW9 | CY0163 | 0.0000 | 0.0488 |
| Pol_592-599_QP8 | CY0163 | 0.0094 | 0.0102 |
| Rev_59-68_SP10 | CY0163 | 0.0000 | 0.0199 |
| Nef_103-111_RM9 | CY0163 | 0.0000 | 0.0171 |
| Env_620-628_TL9 | CY0164 | 0.0000 | 0.0356 |
| Pol_592-599_QP8 | CY0164 | 0.0000 | 0.0077 |
| Rev_59-68_SP10 | CY0164 | 0.0028 | 0.0246 |
| Nef_103-111_RM9 | CY0164 | 0.0000 | 0.0141 |
| Gag_386-394_GW9 | CY0166 | 0.0043 | 0.0440 |
| Pol_592-599_QP8 | CY0166 | 0.0000 | 0.0250 |
| Rev_59-68_SP10 | CY0166 | 0.0029 | 0.0186 |
| Nef_103-111_RM9 | CY0166 | 0.0069 | 0.0795 |

| **No variation detected by Sanger or pyrosequencing** | | | |
| --- | --- | --- | --- |
| Epitope | Animal | π_S_ | π_N_ |
| Gag_459-467_TV9 | CY0163 | 0.0161 | 0.0000 |
| Tat_59-67_CF9 | CY0163 | 0.0000 | 0.0026 |
| Gag_221-229_PR9 | CY0163 | 0.0123 | 0.0037 |
| Env_620-628_TL9 | CY0163 | 0.0000 | 0.0011 |
| Gag_28-37_KA10 | CY0164 | 0.0000 | 0.0000 |
| Gag_459-467_TV9 | CY0164 | 0.0054 | 0.0000 |
| Tat_59-67_CF9 | CY0164 | 0.0000 | 0.0000 |
| Gag_146-154_HL9 | CY0164 | 0.0733 | 0.0000 |
| Gag_221-229_PR9 | CY0164 | 0.0000 | 0.0055 |
| Gag_459-467_TV9 | CY0166 | 0.0000 | 0.0000 |
| Tat_59-67_CF9 | CY0166 | 0.0000 | 0.0031 |
| Gag_221-229_PR9 | CY0166 | 0.0000 | 0.0000 |

* An epitope was considered variant if it had at least one amino acid variant present at a frequency of 1% or greater and it did not match the inoculum.

| **Remainder of genes** | | | |
| --- | --- | --- | --- |
| Gene | Animal | π_S_ | π_N_ |
| *gag* | CY0163 | 0.0066 | 0.0015 |
| *pol* | CY0163 | 0.0067 | 0.0014 |
| *rev* | CY0163 | 0.0018 | 0.0088 |
| *nef* | CY0163 | 0.0097 | 0.0103 |
| *env* | CY0163 | 0.0122 | 0.0039 |
| *tat* | CY0163 | 0.0110 | 0.0040 |
| *gag* | CY0164 | 0.0066 | 0.0017 |
| *pol* | CY0164 | 0.0097 | 0.0009 |
| *rev* | CY0164 | 0.0073 | 0.0068 |
| *nef* | CY0164 | 0.0032 | 0.0075 |
| *env* | CY0164 | 0.0137 | 0.0055 |
| *tat* | CY0164 | 0.0051 | 0.0041 |
| *gag* | CY0166 | 0.0099 | 0.0013 |
| *pol* | CY0166 | 0.0107 | 0.0020 |
| *rev* | CY0166 | 0.0025 | 0.0079 |
| *nef* | CY0166 | 0.0053 | 0.0052 |
| *env* | CY0166 | 0.0145 | 0.0057 |
| *tat* | CY0166 | 0.0080 | 0.0030 |
